# Perceptual Fading of a Stabilized Cortical Image: Replication in the Undergraduate Classroom

**DOI:** 10.1523/ENEURO.0323-21.2021

**Published:** 2021-10-08

**Authors:** Nicole B. Massa, Jacob H. Deck, Michael A. Grubb

**Affiliations:** Department of Psychology and Neuroscience Program, Trinity College, Hartford, CT 06106

**Keywords:** active learning, adaptation, contrast, undergraduate teaching

## Abstract

Prolonged exposure to a stimulus causes desensitization of cortical neurons and results in perceptual changes. One example of this phenomenon is contrast adaptation, in which perceived differences between light and dark regions of a stimulus decrease. Blakemore, Muncey, and Ridley reported evidence for the “perceptual fading of a stabilized cortical image” in a 1971 *Nature* paper. Our goal was to replicate their second experiment, in which adaptation was measured across many contrasts, and develop an active learning exercise for undergraduate students. The experiment was coded using an open-source python package and psychophysical data were collected from two observers. On each trial, a sinusoidally modulated luminance grating appeared above fixation, and the task of the observer was to adjust the contrast of a grating below fixation until the two appeared identical. Between trials in the adaptation condition, a high contrast grating was presented in the top location; no such grating appeared between trials in the control condition. Contrast matches showed a clear reduction during the adaptation condition, thus demonstrating perceptual fading and a successful replication of Blakemore et al. (1971). We then simplified the approach and modified the code to create a single, seamless experience for use in the classroom. With instructions and theoretical background provided in a one-page handout, students can perform the experiment on themselves and view their results in an automatically generated figure. This exercise, a primary example of active learning, will help students gain a first-hand understanding of the perceptual effects of adaptation.

## Significance Statement

Adaptation, a foundational concept in neuroscience, is a reduction in the neural response to frequent stimuli. Adaptation was reported in a 1971 *Nature* paper by Blakemore, Muncey, and Ridley. Here, we replicated their seminal work on adaptation using a free, open-source Python package. On each trial, observers kept their eyes on a central fixation point and adjusted the contrast of a lower grating until it looked identical to that of an upper grating. Contrast matches were made after prolonged exposure to a high-contrast grating in the upper location (adaptation condition), and when no such grating was presented (control condition). We then streamlined the experimental approach and created an active learning exercise intended for students in undergraduate neuroscience courses.

## Introduction

Selective adaptation is the process by which prolonged exposure to a stimulus causes desensitization of cortical neurons, resulting in perceptual changes (Goldstein and Brockmole, 2016; Snowden et al., 2012). Adaptation is critical for efficient neural coding, and its effects have been well characterized at the psychological and neural levels (for review, see Clifford, 2002; Krekelberg et al., 2006; Webster, 2011). Adaptation aftereffects are ubiquitous in the sensory domain and lead to marked biases in perception. Judging the orientation of a line, for example, is systematically biased by first viewing a line of differing orientation (Gibson and Radner, 1937). Analogous results have been reported for motion direction (Levinson and Sekuler, 1976), velocity (Goldstein, 1957), and even high-level facial attributes like gender (Webster et al., 2004) and attractiveness (Rhodes et al., 2003).

Seminal work on the effects of adaptation on perceived contrast was reported in *Nature* by Blakemore et al. (1971). In their foundational experiments, they used two oscilloscope screens, separated by a luminous horizontal fixation bar, to display an “upper” and “lower” grating stimulus. Blakemore and Muncey both acted as observers. Their task was to adjust the lower grating’s contrast to match that of the upper grating using a logarithmic potentiometer. In their experiment 1, the upper grating was presented at two contrast levels (0.7, 0.32) and prolonged exposure to a high contrast grating in the upper location led to a reduction in apparent contrast for both test contrasts. This effect was observed within ∼30 s of initial exposure and reached maximal magnitude within ∼180 s. In their experiment 2, various contrast levels were used, the contrast of the upper test grating varied from trial to trial, and matches were made under two conditions: adaptation and control. The adaptation condition began with 180 s of fixation during which a high contrast grating (0.7) was presented at the upper location. Between adaptation trials, the high contrast upper grating was displayed for 10 s to maintain adaptation. During the control condition, no high contrast grating was presented at the beginning of the condition or between trials. The resulting data indicated (1) perceptual fading occurred as a result of adaptation (i.e., the lower grating needed less contrast to match the perceived contrast of the upper grating), and (2) this effect was more pronounced at lower contrasts. Here, our goal was to replicate Blakemore, Muncey, and Ridley’s experiment 2 and to develop an active learning exercise for use in undergraduate psychology and neuroscience courses.

As the name implies, active learning requires that students actively participate in the process of knowledge acquisition, rather than passively receive it (Bonwell and Eison, 1991). Ample empirical evidence confirms that student engagement in the learning process results in improved recall and greater conceptual understanding (for review, see Prince, 2004). In an oft-cited example from physics education with over 6000 high-school and college students, Hake found that students in classes that promoted interactive-engagement methods, compared with students in traditional lecture-based classes, showed significantly greater improvement from pre-test to post-test (Hake, 1998). Similarly, Yoder and Hochevar (2005) found that psychology students’ performance on multiple-choice examination items was better when the question’s material had been covered with active learning techniques compared with other formats.

With the active learning exercise provided in this article, we hope to similarly improve students’ conceptual understanding of adaptation. We provide a one-page handout for use as homework assignment or (time-permitting) in-class activity, with links to the necessary open-source PsychoPy code and detailed instructions for downloading and running the experiment. During this ∼45-min active learning exercise, students will be able to observe evidence of contrast adaptation within their own visual systems and deepen their understanding of this foundational concept.

## Materials and Methods

### Replication of Blakemore et al. (1971)

#### Observers

Two undergraduate researchers (one woman, one man), aged 19, who are also authors of this manuscript, volunteered to participate in the study. Both were assigned the same experimental task. Informed consent was obtained from both observers and procedures were approved by the Trinity College Institutional Review Board.

#### General procedure

In both experimental conditions (i.e., adaptation, control), the observer maintained fixation for the duration of the experiment. Using the computer mouse to control an interactive slider, the observer attempted to match the perceived contrast of the lower grating to that of the upper grating as quickly and accurately as possible. Once a contrast match had been made, they pressed “d” on the keyboard to submit the response.

#### Stimuli

On a full screen, mid-gray background, two vertical sinusoidally-modulated luminance gratings enveloped in a Gaussian mask [spatial frequency, three cycles per degree; size, 3 × 3 degrees of visual angle (DVAs)] were positioned three DVA above and below a central fixation point (white +, 0.5 × 0.5 DVA). A white line representing an interactive slider was positioned below the lower grating. Use of this slider dynamically changed the contrast of the lower grating. The scale had 1000 incremental steps; each incremental step corresponded to a contrast from a contrast list. The contrast list consisted of 1000 values between 0.01 and 1.0 that were spaced evenly on a logarithmic scale. The value zero was added as the first number of the contrast list and corresponded with the first position of the interactive slider, thus allowing the lower grating to be effectively invisible. Each subsequent position of the slider was paired with the respective contrast level. Both the replication of Blakemore et al. (1971) and the active learning exercise were programmed in PsychoPy (Peirce and MacAskill, 2018).

#### Control condition

##### Stimuli

The upper grating was pseudo-randomly selected to be 1 of 11 contrast values on each trial: 0.0200, 0.0285, 0.0407, 0.0581, 0.0829, 0.1183, 0.1688, 0.2409, 0.3438, 0.4906, and 0.7000. These values were approximated from Blakemore et al. (1971; their Fig. 1).

**Figure 1.**
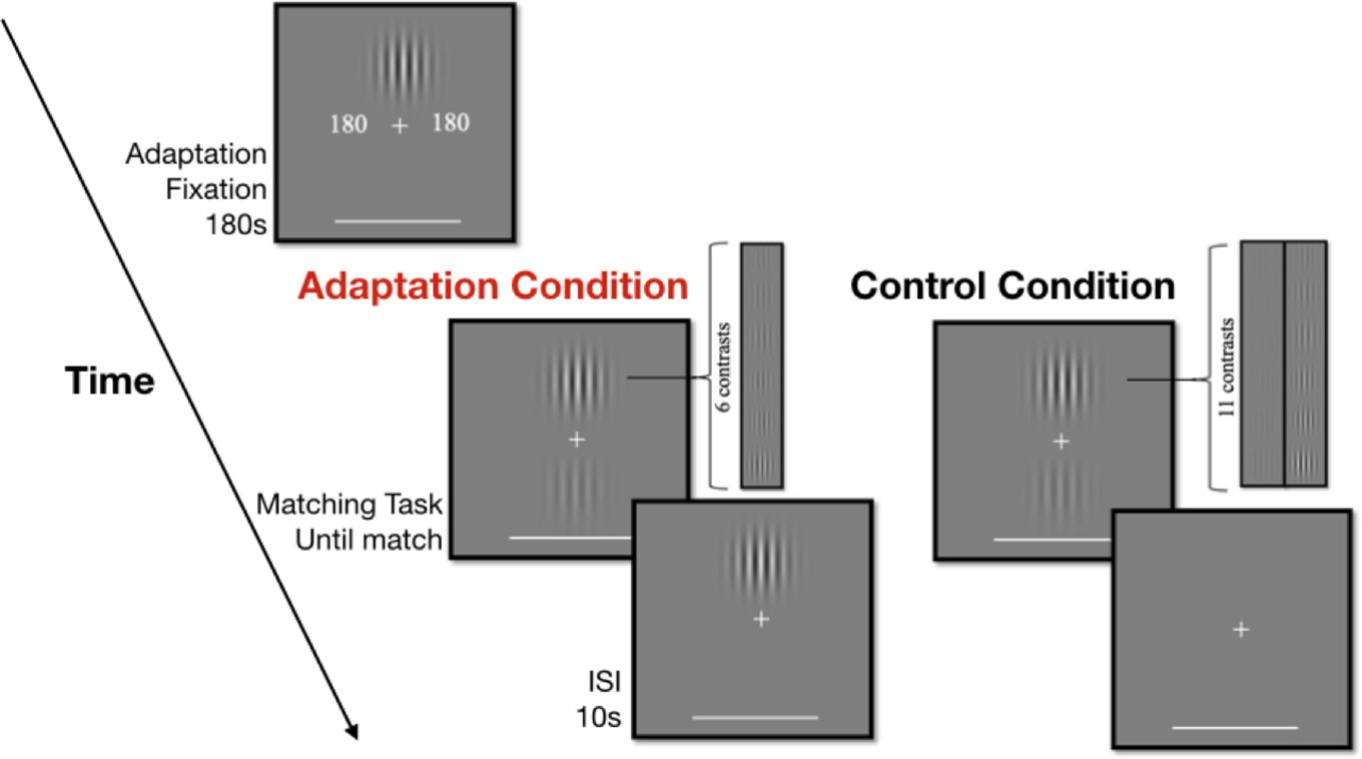
Blakemore et al. (1971) replication procedure. The adaptation condition began with 180 s of adaptation. Between each trial was a pause of 10 s, during which a high contrast grating was presented at the upper location in the adaptation condition; no such grating was shown between trials in the control condition. The contrast of the upper grating was randomly determined on each trial; 10 contrast matches were made for each contrast level within each condition. ISI, inter-stimulus interval.

##### Procedure

On each trial, the observer matched the perceived contrast of the lower grating to that of the upper grating while maintaining fixation (Fig. 1). Between each trial was a 10-s pause in which the observer was still expected to maintain fixation. There were 110 trials for each session (10 repetitions of each contrast).

#### Adaptation condition

##### Stimuli

The upper grating was pseudo-randomly selected to be 1 of 6 contrast levels for each trial: 0.1183, 0.1688, 0.2409, 0.3438, 0.4906, and 0.7000. These values were approximated from Blakemore et al. (1971; their Fig. 1).

##### Procedure

Before the trials began, there was a 180-s period in which the observer maintained fixation while a countdown timer (to the left and right of fixation) and an upper grating with a contrast of 0.7 were displayed (Fig. 1). Following this initial period of adaptation, the observer matched the perceived contrast of the lower grating to that of the upper grating while maintaining fixation on each trial. Between trials, this condition differed from the control condition in that a 0.7 contrast upper grating was displayed for 10 s. There were 60 trials for each session (10 repetitions of each contrast).

#### Data analysis and statistics

Following the analysis reported in Blakemore et al. (1971), we took the mean across contrast matches (10 repetitions) made for each contrast level, separately for each condition (control, adaptation) and separately for each observer. We then used linear regression to fit a straight line to log-transformed data (pooling across observers as in Blakemore et al., 1971), separately for each condition.

As a complement to the Blakemore et al. (1971) replication analysis, we also fit a linear mixed effects model to all of the (non-averaged) log-transformed data simultaneously (Table 1, Eq. 1). Upper grating contrast, experimental condition (0 = control, 1 = adaptation), and a contrast X experimental condition interaction were included as fixed-effects; observer ID was included as a random effect. The two participant ID numbers (1 and 2) were included as random effects in the analysis so that the y-intercept of the regression line could be estimated separately for each observer, thus allowing differences in matched contrasts based on each individual’s unique visual system. Analyses were completed in MATLAB (The MathWorks, 2020).

(1; linear mixed effects model) $log(contrastMatch)=\beta_{0}+\beta_{1}*log(upperContrast)+\beta 2*isAdaptation+\beta_{3}*log(upperContrast)*isAdaptation+(1|ID).$

**Table 1:** Statistical summary

Data structure	Type of test	Power
Normal distribution	Linear mixed effects model	95% CIs

#### Apparatus

The experiment was conducted in the same dark, silent room with a headrest used to keep the distance and height from the screen the same for every session and observer. The same iMac (21.5-inch, 2017), 2.3 GHz Dual-Core Intel Core i5, 8 GB 2133 MHz DDR4, Intel Iris Plus Graphics 640 1536 MB computer was used for all sessions of the experiment, with the screen brightness set to the highest setting.

#### Active learning exercise

##### Observers

The same two observers from the Blakemore et al. (1971) replication experiment completed the active learning exercise.

##### Procedure

**Figure 2.**
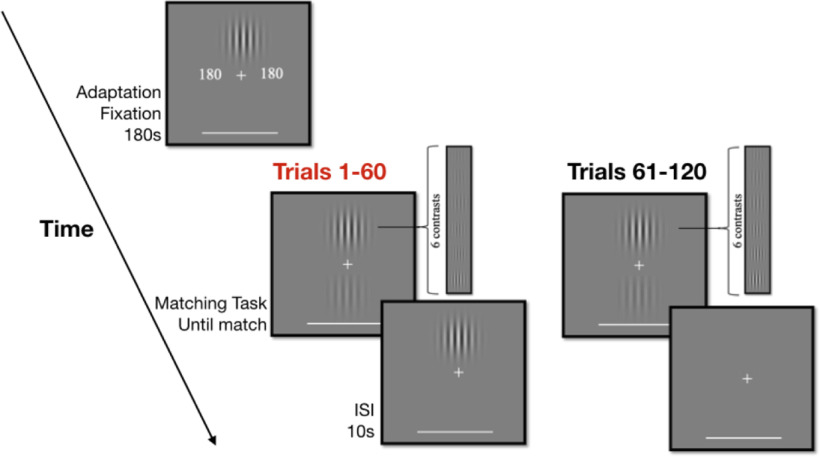
Active learning exercise procedure. The adaptation condition began with 180 s of adaptation. Between each trial was a pause of 10 s, during which a high contrast grating was presented at the upper location in the adaptation condition; no such grating was shown between trials in the control condition. The contrast of the upper grating was randomly determined on each trial; 10 contrast matches were made for each contrast level within each condition. ISI, inter-stimulus interval.

The observers first completed 6, non-recorded practice trials, similar in structure to the control trials of the replication experiment. Upon further instruction, they began 60 control trials (10 repetitions for each contrast). On each trial, the observer matched the perceived contrast of the lower grating to that of the upper grating while maintaining fixation (Fig. 2). Between each trial was a 10-s pause in which the participant was still expected to maintain fixation. After the control condition, the participant had the option to take a break before the 180 s adaptation period. During the adaptation period, the participant maintained fixation while a countdown timer (to the left and right of fixation) and an upper grating with a contrast of 0.7 were displayed (Fig. 2). Following this initial period of adaptation, the participant did the same matching task during the 60 adaptation trials (10 repetitions for each contrast). Between trials, this condition differed from the control condition in that a 0.7 contrast upper grating was displayed for 10 s. A figure displaying the data and lines of best fit for the average matched contrasts as a function of the top contrast for each condition was automatically created and saved to the observer’s computer (see Fig. 5), as was a CSV file with the raw data used to make the figure.

##### Stimuli

The adaptation and control conditions used the same six contrast levels from our replication experiment (0.1183, 0.1688, 0.2409, 0.3438, 0.4906, and 0.7000). All contrasts levels were used an equal number of times, and the level was random determined on each trial.

**Figure 3.**
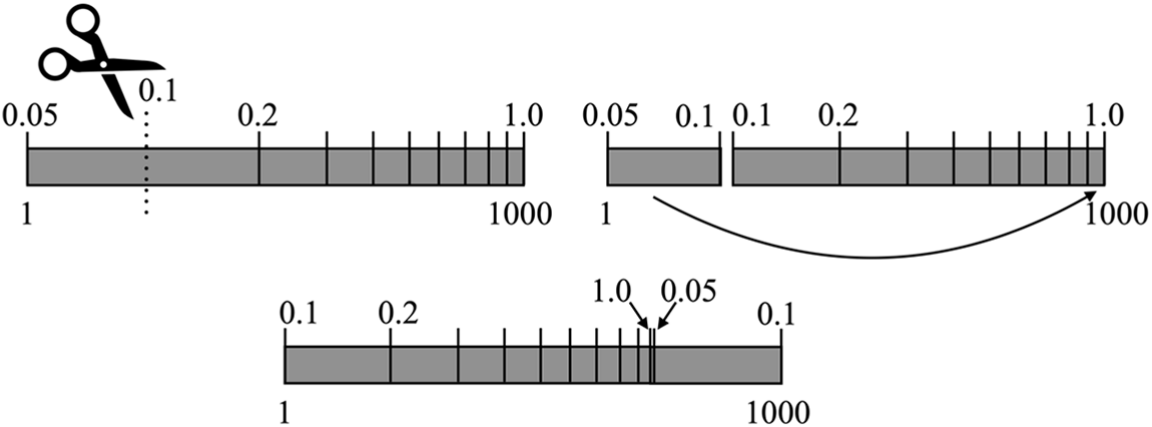
Sliding scale. A visual representation of the change to the sliding scale (top numbers, contrast levels; bottom numbers, position values). At a random position value, the logarithmic scale was cut. The contrast of 0.1, which corresponded to the cut position value became the beginning of the sliding scale. All contrasts before this cut were added to the end of the scale.

To prevent any potential undue influence of the interactive slider’s settings on an untrained observer’s judgements, we modified the slider for the active learning exercise. The scale still had 1000 incremental steps, with each incremental step corresponding to a contrast from a contrast list. The contrast list consisted of 1000 values between 0.05 and 1.0 that were spaced evenly on a logarithmic scale. Each subsequent position of the slider was paired with the respective contrast level. On each trial, the logarithmic scale was cut at a random point. The contrast level corresponding to this random point became newly paired with the smallest position value. All greater contrasts shifted down in position value; all lesser contrasts were added to the right-most end of the scale (Fig. 3). This forces the observer to actually sample the slider scale on each trial, rather than allowing them to rely on memories of settings used in former trials.

### Code accessibility

The data and analysis code described in the paper are freely available online at https://attentionperceptiondecision.com/adaptation/. Both are available as Extended Data 1 and Extended Data 2.

10.1523/ENEURO.0323-21.2021.ed1 Extended Data 1 Replication analysis code and data. Download Extended Data 1, ZIP file.

10.1523/ENEURO.0323-21.2021.ed2 Extended Data 2 Active learning exercise code and instructions. Download Extended Data 2, ZIP file.

## Results

### Replication of Blakemore et al. (1971)

**Figure 4.**
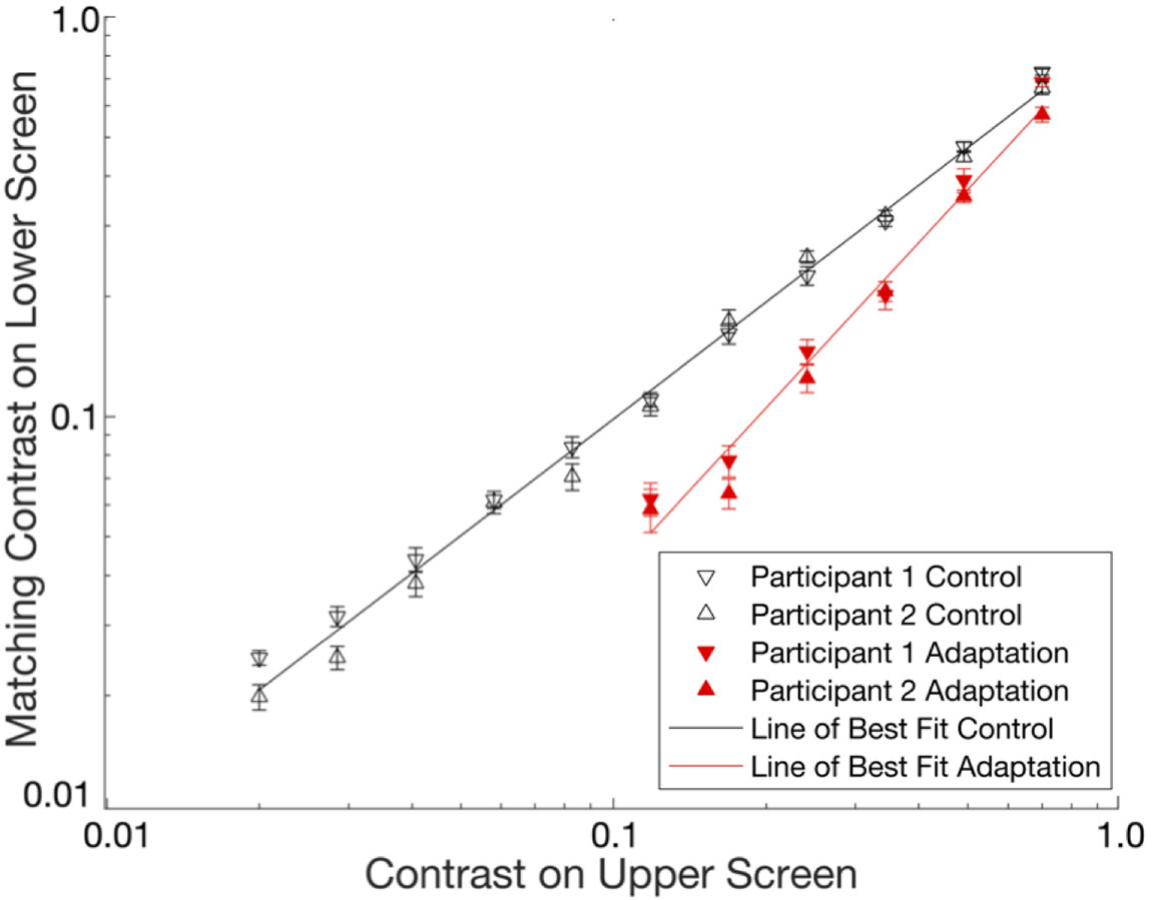
Blakemore et al. (1971) replication results. Perceived contrast as a function of physical contrast. Each point represents the average of ten matches (open triangles, control condition; red triangles, adaptation condition). Error bars, standard error of the mean.

We replicated the three critical findings reported in Blakemore and colleagues’ experiment 2 (Fig. 4). First, the slope of the best-fitting line for contrast matches made during the control condition was effectively +1 [slope = 0.97174, 95% confidence interval (CI) = [0.9380–1.0055]; Table 2]. Second, adaptation to a high contrast grating altered perceived contrast: relative to the control condition, average contrast matches during adaptation were reduced at all tested contrasts (Table 3). Third, this reduction in apparent contrast was more pronounced at lower contrast levels: the slope of the best-fitting line for contrast matches made during adaptation was greater than +1 (slope = 1.3746, 95% CI = [1.2295–1.5197]; Table 2).

**Table 2:** Simple linear models

Model information	Number of observations	Error degrees of freedom	Root mean squared error	R^2^	Adjusted R^2^	F statistic vs constant model	p value
Control condition	22	20	0.0853	0.994	0.994	3.61E + 03	4.52E-24
Adaptation condition	12	10	0.137	0.978	0.976	445	1.27E-09
Estimated coefficients	Estimate	SE	95% CI			t statistics	p value
Control condition							
Intercept	−0.07842	0.039006	[−0.1598, 0.0029]			−2.0105	0.05806
log(upperContrast)	0.97174	0.016167	[0.9380, 1.0055]			60.106	4.5188E-24
Adaptation condition							
Intercept	−0.039806	0.090256	[−0.2409, 0.1613]			−0.44104	0.66857
log(upperContrast)	1.3746	0.065132	[1.2295, 1.5197]			21.105	1.2679E-09

Full results. Data and analysis code are available as Extended Data 1. SE, standard error; CI, confidence interval; DF, degrees of freedom.

**Table 3:** Contrast match data

Upper contrast	Observer 1 contrast matches				Observer 2 contrast matches			
	Control condition		Adaptation condition		Control condition		Adaptation condition	
0.0200	0.0248	(0.0046)			0.0198	(0.0064)		
0.0285	0.0316	(0.0081)			0.0249	(0.0074)		
0.0407	0.0438	(0.0137)			0.0382	(0.0124)		
0.0581	0.0618	(0.0136)			0.0610	(0.0174)		
0.0829	0.0839	(0.0230)			0.0707	(0.0238)		
0.1183	0.1105	(0.0198)	0.0622	(0.0264)	0.1061	(0.0249)	0.0585	(0.0324)
0.1688	0.1614	(0.0422)	0.0775	(0.0314)	0.1734	(0.0512)	0.0641	(0.0245)
0.2409	0.2251	(0.0536)	0.1457	(0.0461)	0.2510	(0.0399)	0.1247	(0.044)
0.3438	0.3088	(0.0438)	0.2013	(0.0718)	0.3186	(0.0438)	0.2059	(0.0531)
0.4906	0.4746	(0.0535)	0.3906	(0.1204)	0.4467	(0.0563)	0.3563	(0.0579)
0.7000	0.7282	(0.0601)	0.6866	(0.0798)	0.6638	(0.1069)	0.5698	(0.1082)

Each observer’s mean (SD in parentheses) of 10 matches for each upper contrast in the control and adaptation conditions.

A linear mixed effects model that fit all data simultaneously and included observer as a random effects parameter provided converging results (Table 1): the upper contrast × adaptation condition interaction was significantly greater than zero and its 95% CI excluded zero as a potential value (estimate = 0.44 263, *p* = 1.0013e-36, 95% CI = [0.37 803–0.50 724]; see Table 4 for full reporting).

**Table 4:** Linear mixed effects model

Model information	Number of observations	Fixed effects coefficients		Random effects coefficients		Covariance parameters
	680	4		2		2
Model fit statistics	AIC	BIC		LogLikelihood		Deviance
	250.85	277.98		−119.42		238.85
Effect	Estimate	SE	95% CI	t statistics	DF	p value
Fixed effects						
Intercept	−0.084	0.042	[−0.167, −0.002]	−2.0019	676	0.045699
Condition (0 = control, 1 = adaptation)	0.052	0.052	[−0.049, 0.153]	1.0036	676	0.31595
log(upperContrast)	0.982	0.012	[0.958, 1.006]	80.523	676	0
Condition:log(upperContrast)	0.443	0.033	[0.378, 0.507]	13.453	676	1.0013E-36
Random effects						
ID	0.0424		[0.013955, 0.12908]				
Error	0.2875		[0.27262, 0.30324]			

Full results. Data and analysis code are available as Extended Data 1.

### Active learning exercise

**Figure 5.**
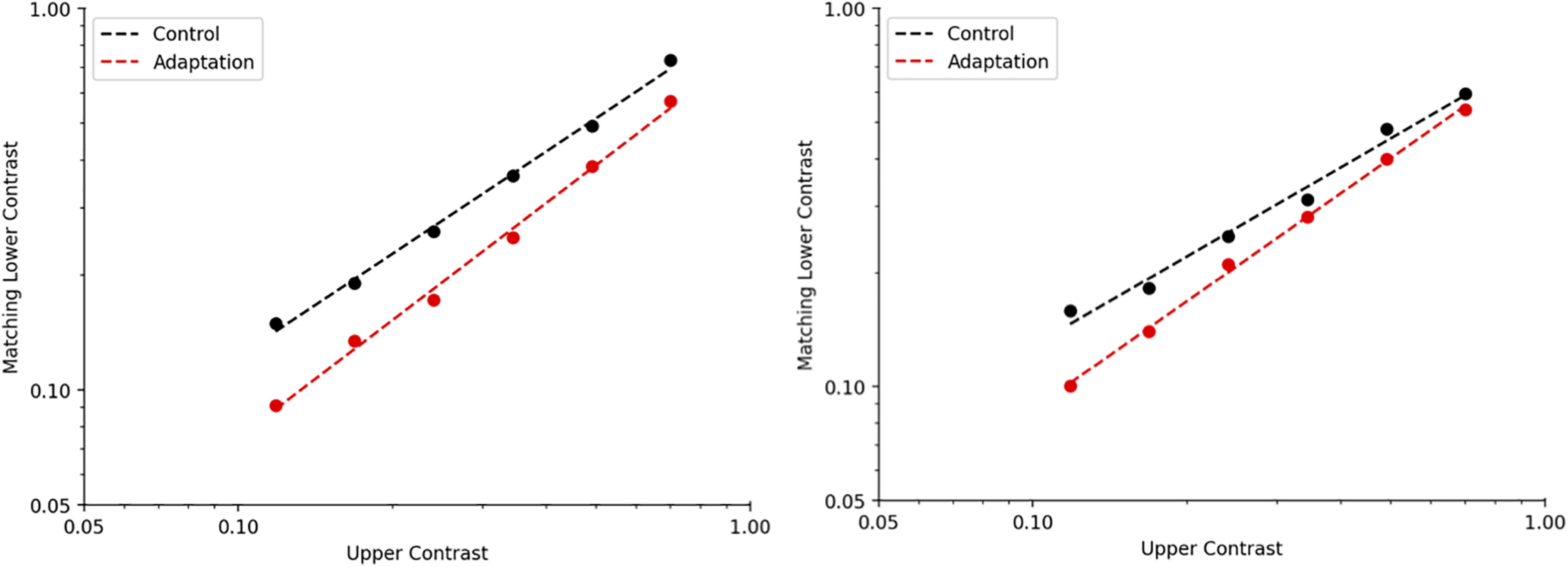
Active learning exercise results. Perceived contrast as a function of physical contrast for two example observers. Each point represents the average of ten matches (black dots, control condition; red dots, adaptation condition).

The primary result of Blakemore, Muncey, and Ridley’s experiment 2 was replicated for both observers in the active learning exercise. For all contrast levels, average contrast matches during adaptation trials were reduced relative to control trials (Fig. 5).

## Discussion

The purpose of this study was to replicate the second experiment of Blakemore et al. (1971), in which adaptation was measured across many contrast levels, and to develop an interactive activity on adaptation for undergraduate students. The results of the replication experiment strongly supported Blakemore, Muncey, and Ridley’s findings that adaptation leads to a decrease in perceived contrast and that this decrease is more apparent at lower contrast levels. The active learning exercise produced converging results.

**Figure 6.**
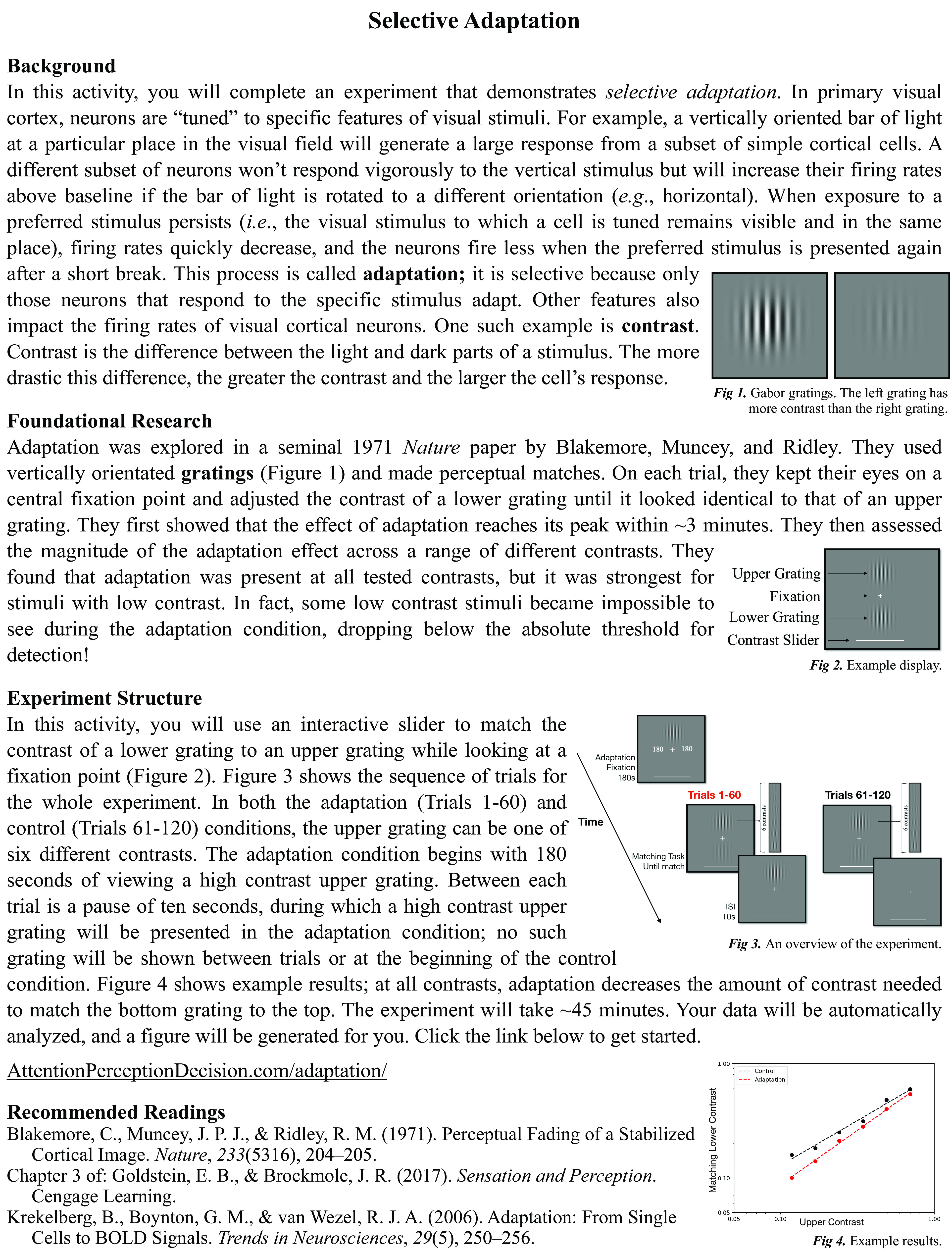
Active learning exercise handout. Experiment code and download instructions available as Extended Data 1.

We envision the active learning exercise being implemented in undergraduate neuroscience and psychology classes that cover adaptation. Using our open-source experiment code, students can collect psychophysical data on themselves, have those data automatically analyzed, and review an auto-generated figure. Professional testing equipment is not required. The ∼45-min activity could be assigned for homework or form part of a learning plan in a longer classroom session (e.g., a lab). Students will first read a one-page handout (Fig. 6) that provides background on perceptual adaptation, summarizes Blakemore, Muncey, and Ridley’s foundational study, and introduces the structure of the active learning activity. The handout contains a URL where students can find detailed instructions on how to download PsychoPy and run the experimental code. Once finished, a CSV file containing the raw data and a figure visualizing the results (Fig. 5) will be saved to the student’s computer allowing for immediate discussion of the outcome. As an advanced extension to this activity, students can use the CSV file to analyze the raw data and recreate the automatically-generated figure.

Additionally, this activity can be used to prompt a discussion on psychophysical experiment classification, specifically, Brindley’s “Class A” and “Class B” distinction. Class A experiments are comprised of trials with correct and incorrect answers, resulting in quantifiable data that can be used to understand the physiological mechanisms involved in perception (Brindley, 1960; Morgan et al., 2013; Kingdom and Prins, 2016). In contrast, Class B experiments focus on the subjective qualities of the stimuli and contextual impacts on appearance. In the case of adaptation, Class B experiments can be powerful and instructive (e.g., after adapting to a green stimulus, a red afterimage will appear on a white surface), but they lack the objectivity of the contrast matching approach used in Blakemore, Muncey, and Ridley’s study and replicated here. After discussing the distinction between these two classes of psychophysical experiments, the discussion can transition to linking these neuroscience methods to epistemology (i.e., how we know what we know) and philosophy of mind.

Two of the authors of this paper, who are undergraduate students themselves, are confident that these kinds of interactive activities will be more effective in deepening students’ understanding of the material. This is consistent with the empirical literature on active learning (Prince, 2004), and we hope students will better retain course information that is complemented by this active learning activity. Using our successful experiences as a guide, our active learning activity caters to students who learn better through visuals and experiences. By making the active learning exercise version of our adaption experiment publicly available, we aim to deepen students’ understanding of the foundational topic of adaptation.

## Synthesis

Reviewing Editor: Christine Portfors, Washington State University Decisions are customarily a result of the Reviewing Editor and the peer reviewers coming together and discussing their recommendations until a consensus is reached. When revisions are invited, a fact-based synthesis statement explaining their decision and outlining what is needed to prepare a revision will be listed below. The following reviewer(s) agreed to reveal their identity: Nestor Matthews, Jennifer Steeves.

This active learning undergraduate targeted experiment will provide students with first-hand experience of fundamental vision science concepts that could perhaps be otherwise difficult to understand/appreciate.

Reviewer 1:

A strength of the manuscript -and of Blakemore et al.'s (1971) classic Nature article- is that it demonstrates a relatively objective way to reveal a highly subjective phenomenon. This point can be appreciated within the context of Brindley's (1960; Physiology of the Retina and Visual Pathways, London” Edward Arnold. 14-16, 20, 319) “Class A” vs “Class B” distinction. Class A experiments comprise trials that have correct and incorrect answers, e.g., the magnitude and direction of a luminance-matching error can be verified independently by a light meter. Class B experiments involve reports of percepts. To quote SUNY Optometry's Ben Backus, “there can be no arguing about percepts”. Critically, many demonstrations of sensory adaptation involve participants “only” subjectively reporting their percepts, e.g., adapting to a stimulus that looks green generates a compellingly red perceptual aftereffect. While such color aftereffect demos can demonstrate the principle of sensory adaptation, they lack the objectivity demonstrated in Blakemore et al.'s (1971) classic Nature paper and again in the present manuscript. The authors might consider emphasizing this distinction (Class A vs Class B) in revision. The Class A vs Class B distinction may also provide undergraduate students with an opportunity to link a specific neuroscience methodology (Class A psychophysics) to philosophy (epistemology - how we know what we know). This could set the stage for Neuroscience classroom discussions about the Philosophy of Mind.

Minor Points (in order of appearance within the manuscript)

Page 5 Line 15: The phrase “institutional IRB” feels redundant, as the “I” in “IRB” already represents the word “Institutional”.

Page 9 Lines 7-8: It was not clear to me how to think about “Observer ID” as a random effect.

Page 10 lines 15-16: The use of root-word “task” feels redundant in “...the participant was _tasked_ to do the same matching _task_...”

Page 12 Lines 6 - 9: I absolutely LOVE that the authors cut a random point on the scale and re-positioned it with the smallest value. As the authors note, this minimizes merely motor-memorizing prior scale positions. The cutting and re-positioning promotes comparisons that are perceptual, rather than motor.

Page 13 line 5: I'm confused by the meaning (and the importance) of Table 1's first column, “In-Text Letter” ('a').

Page 19 Lines 19-21: Two of the authors (who identify as undergraduates) offer a self-report that they find greater comprehension from interactive activities than from more traditional lecture-based structures. The self-report feels (epistemically) a bit out of place for eNeuro. Also, the self-report would be more persuasive if there were a clear control for time spent on the interactive activity and the traditional lecture approach. Spending ∼45 minutes on the proposed exercise would need to be compared to spending ∼45 minutes of lecture on the principle of sensory adaptation.

Reviewer 2:

On the whole, this is an excellent replication of part of a classic paper in vision science that demonstrates a fundamental property of vision and other sensory systems and provides an approachable hands-on experience opportunity for undergraduate students. I congratulate the authors for this work. I have relatively minor comments below.

The notion of equally and randomly seems like an oxymoron. Perhaps pseudo-randomly would be more appropriate language.

Figure 1 could be improved for clarity of procedure. Try adopting timeline-like figures which include the ISI and response times with an arrow for time... Likewise for Figure 2.

While this is a phenomenon with robust findings, it would have been valuable to ask naïve observers to participate to explore the ease of use of the tutorial and in particular to assess the “cut” scale use.

Tables 2 and 3 are a bit unusual and perhaps could be added as supplementary material since they simply replicate the data from Figure 4. Also, they should be formatted according to more standard Table formatting such as APA. Table 4 could also be in supplementary materials.

Would it be helpful to give the raw data and ask the students to plot it themselves rather than to provide it in the tutorial? Perhaps this could be an option in this active learning tutorial for use in more senior classes. I would use this option in my senior perception lab course.
