# Erratum: Massa et al., “Perceptual Fading of a Stabilized Cortical Image: Replication in the Undergraduate Classroom”

**DOI:** 10.1523/ENEURO.0161-22.2022

**Published:** 2022-05-25

In the article, “Perceptual Fading of a Stabilized Cortical Image: Replication in the Undergraduate Classroom,” by Nicole B. Massa, Jacob H. Deck, and Michael A. Grubb, which published online on September 16, 2021, there was an error related to the methodology that the authors want to clarify. In the originally published version, a mean of 10 contrast matches was noted throughout the article. Because the 10 contrast matches were done twice, the total should have read as 20. The references to 10 matches have been corrected throughout to 20. This does not affect the conclusions of the paper, and the online version has been corrected.

