# Supplementary material for: Perceptual Fading of a Stabilized Cortical Image: Replication in the Undergraduate Classroom
Source: eNeuro. 2021 Oct 8;8(5):ENEURO.0323-21.2021. doi: 10.1523/ENEURO.0323-21.2021 (PMC8503964; doi:10.1523/ENEURO.0323-21.2021)

Figure 6-2: Active Learning Activity Instruction Manual.

Adaptation Activity Instruction Manual

Mac and Windows Users

1. **Go to https://www.psychopy.org**
2. **Click the install tab at the top right of the screen**

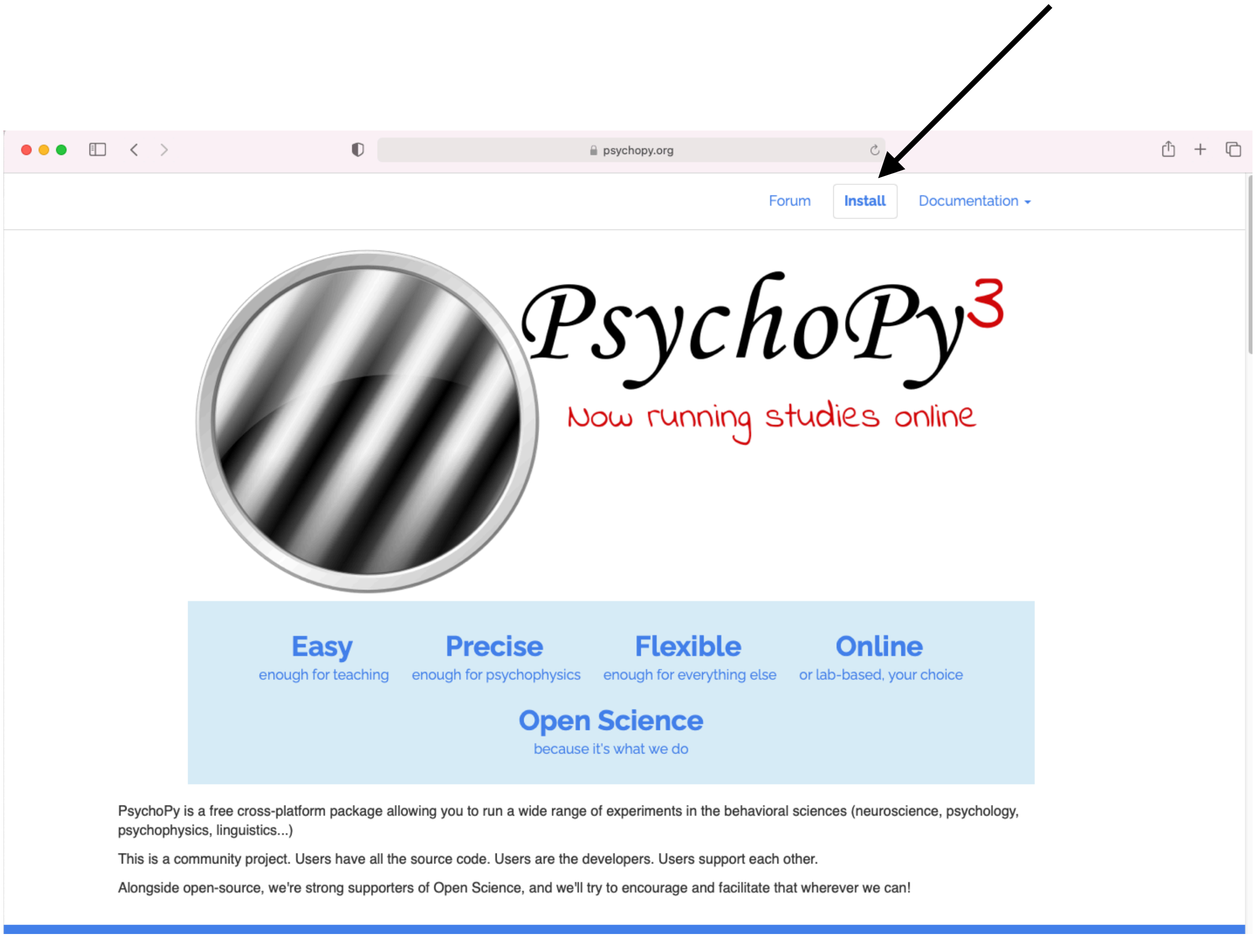

Mac and Windows Users

3. Click the blue box displaying the recommended PsychoPy version to install

Note: PsychoPy will recommend a different version depending on your operating system

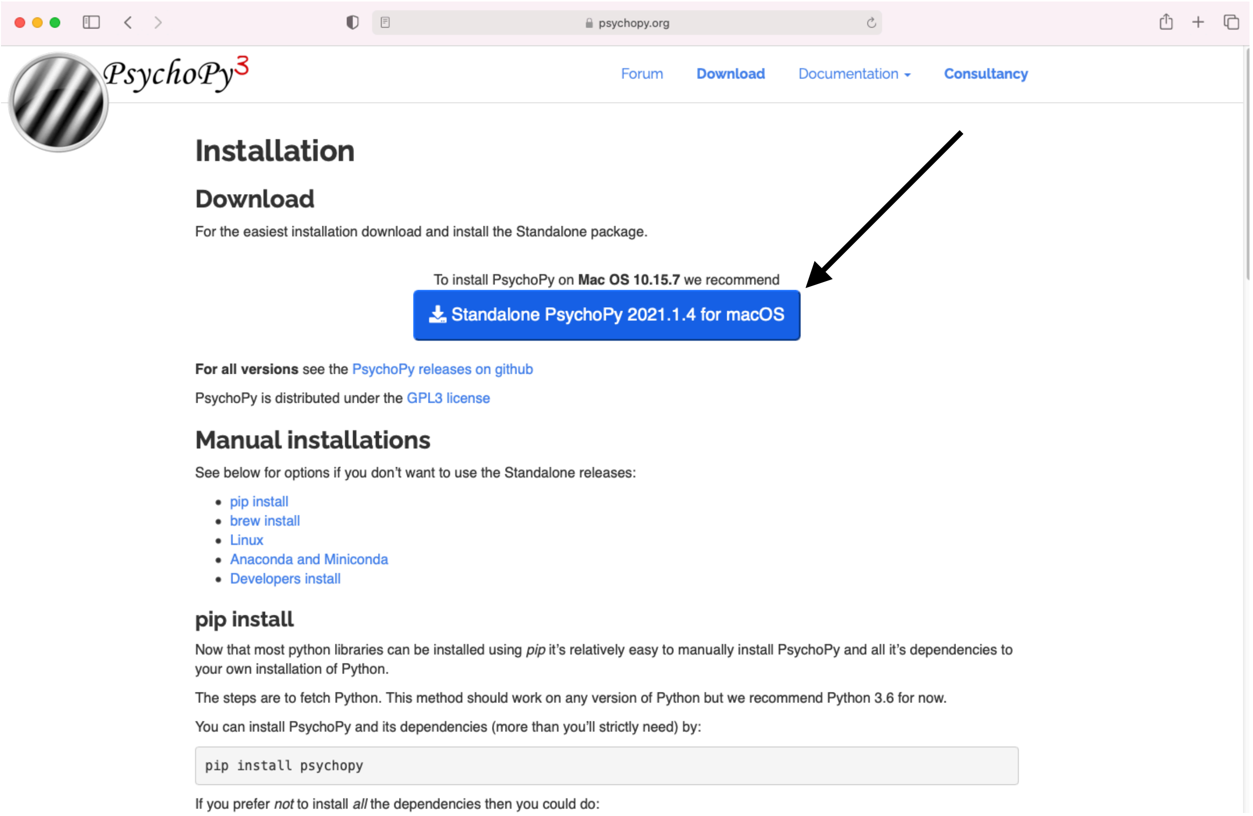

Mac Users

4. **Open Finder and click on applications**
5. **Open PsychoPy application**

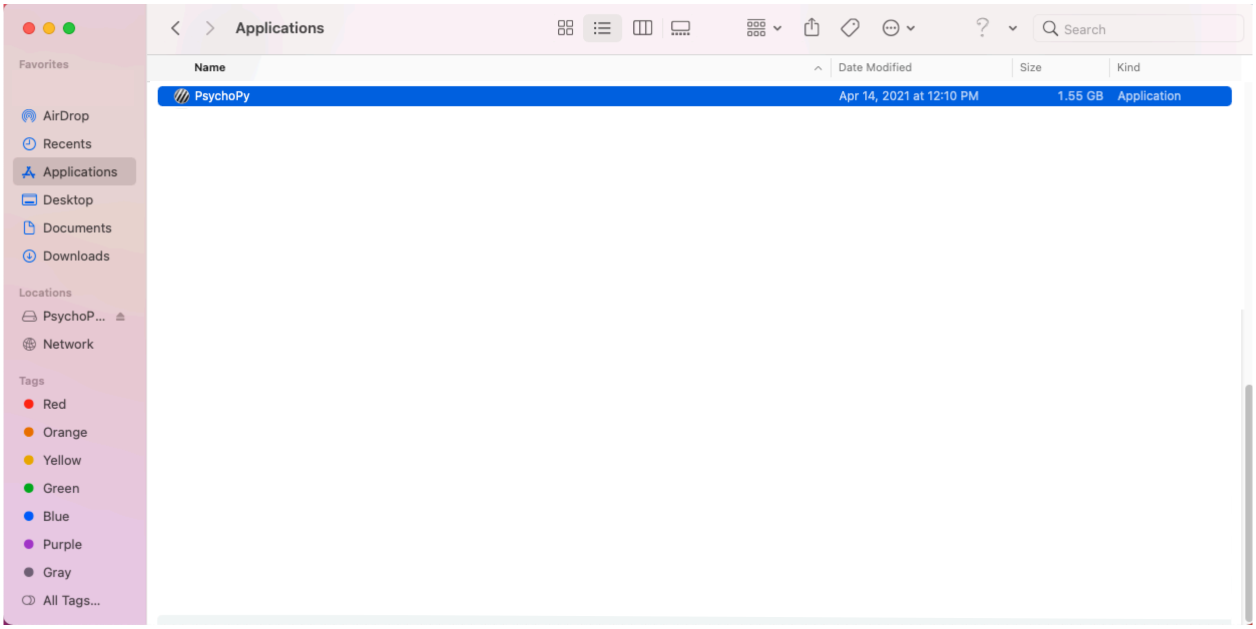

Windows Users

4. Press the windows icon in the bottom left corner
5. Search PsychoPy in the search bar and open the application

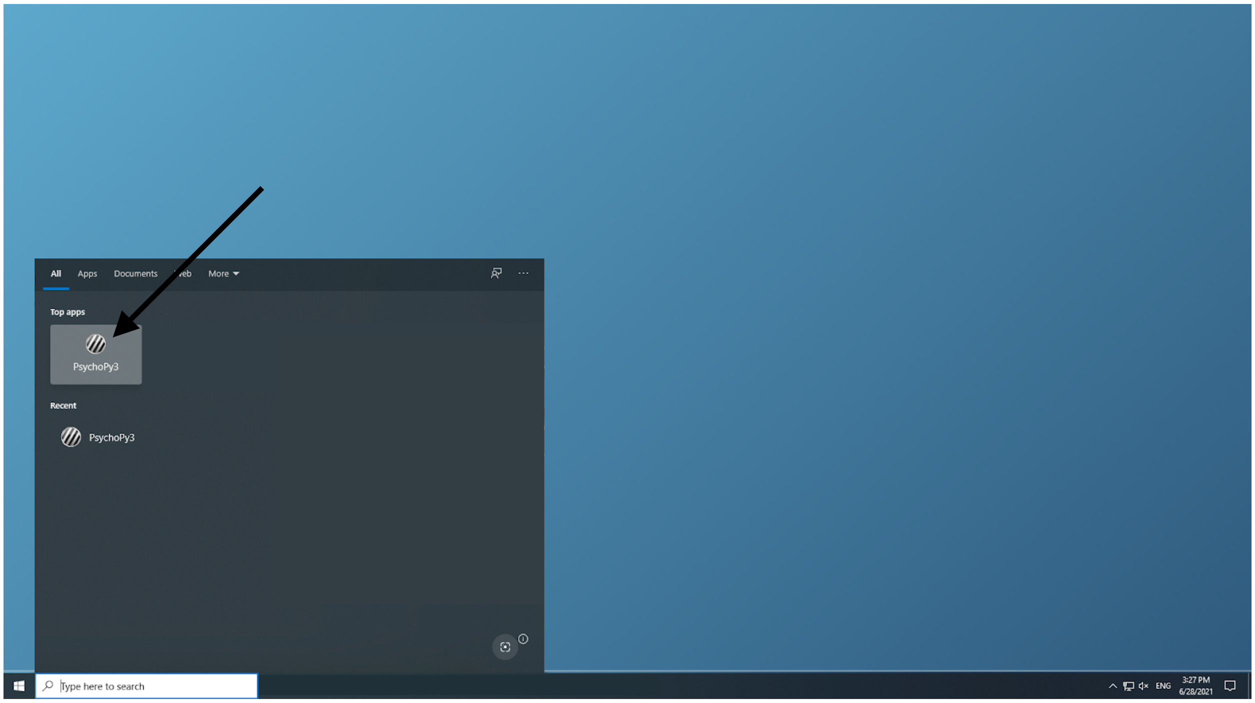

Mac Users

6. **After PsychoPy launches, click on the *Window* tab at the top of your screen and select the option “PsychoPy Coder”**

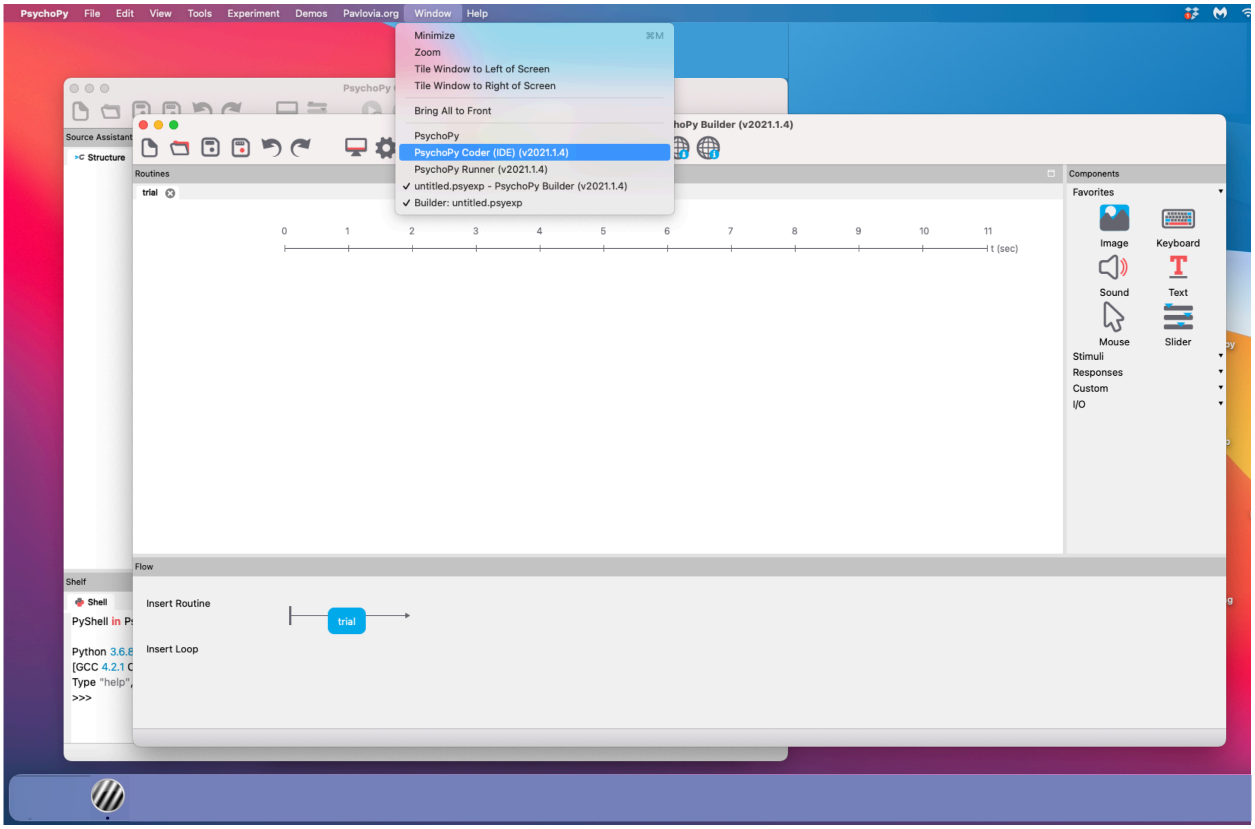

Windows Users

6. After PsychoPy launches, hover over the PsychoPy icon at the bottom of your screen and select the box labeled "PsychoPy Coder"

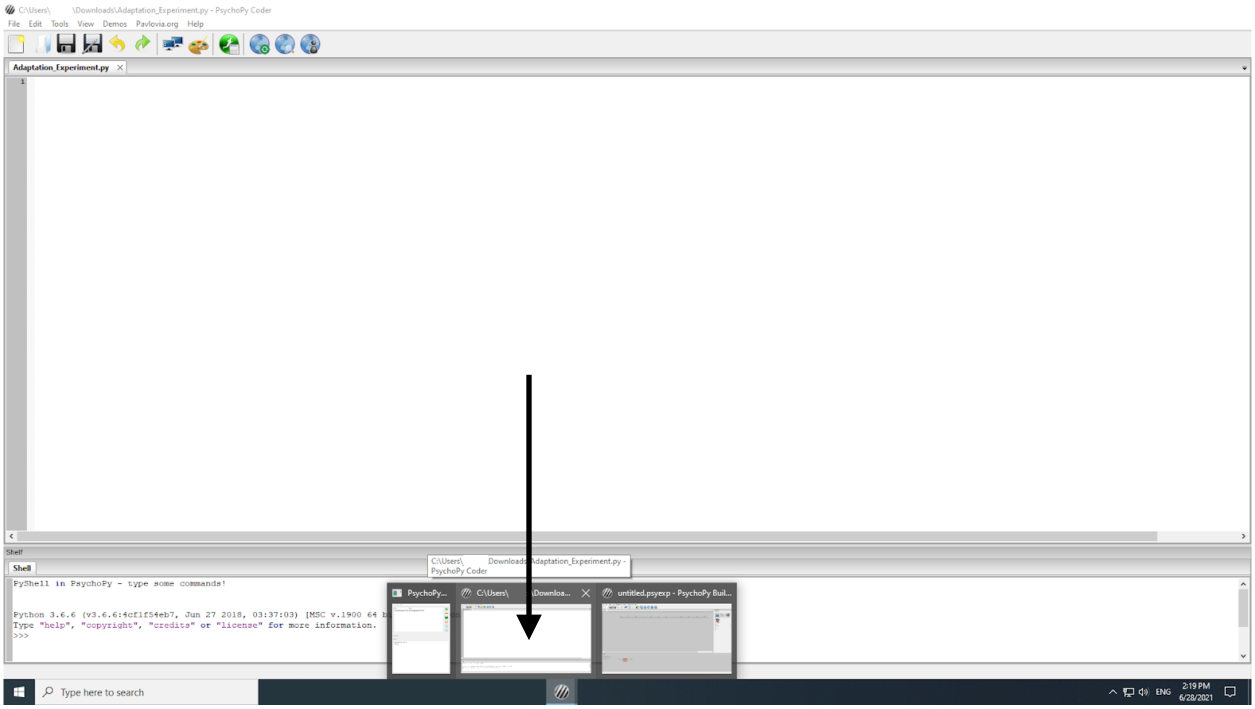

Mac and Windows Users

7. **Download the Adaptation_Experiment file from: (URL redacted for double-blind review)**

Mac and Windows Users

8. In the upper left corner of the screen, click the folder icon

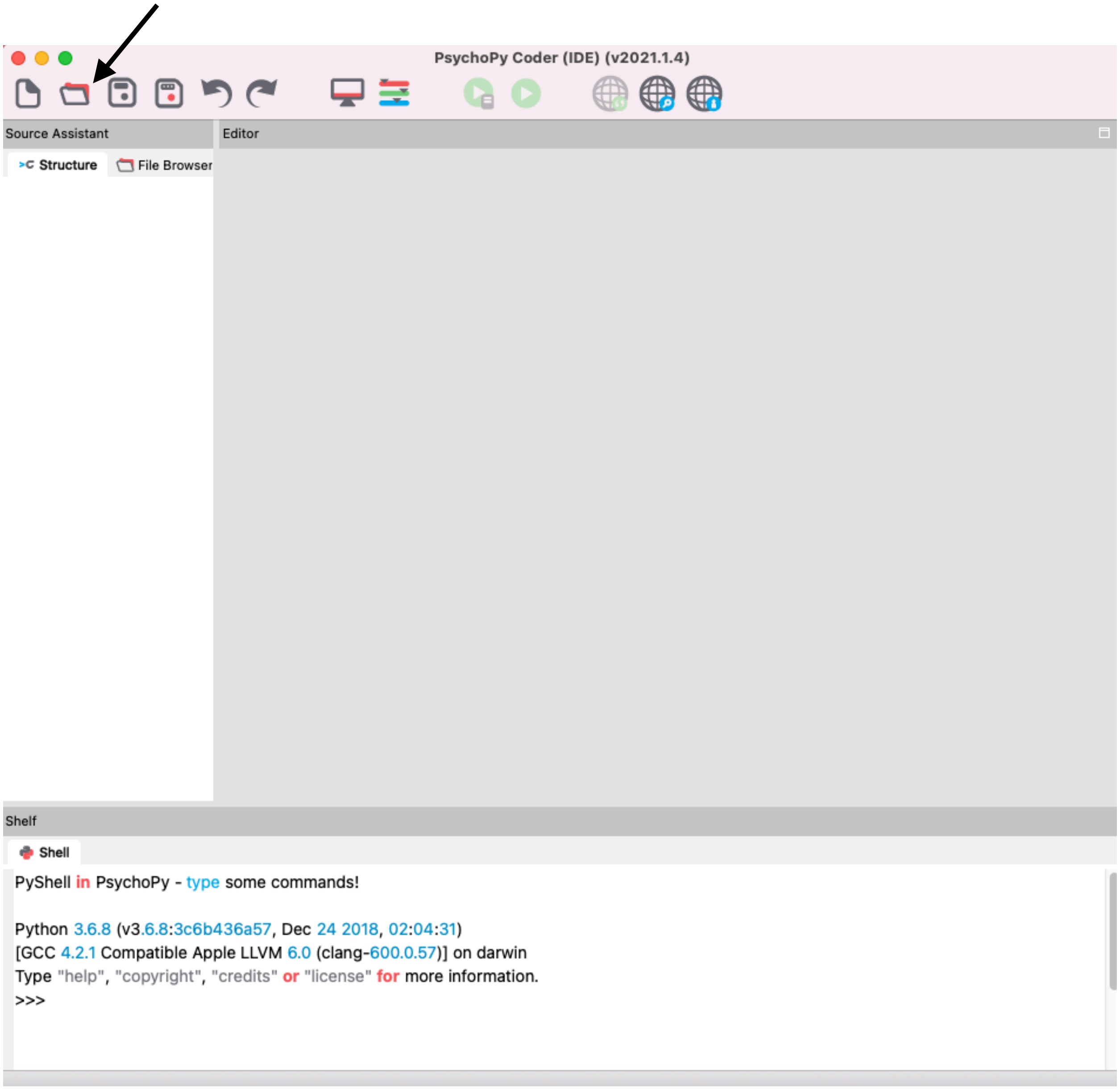

Mac and Windows Users

9. Locate the file “Adaptation_Experiment” in the *Downloads* folder and click open

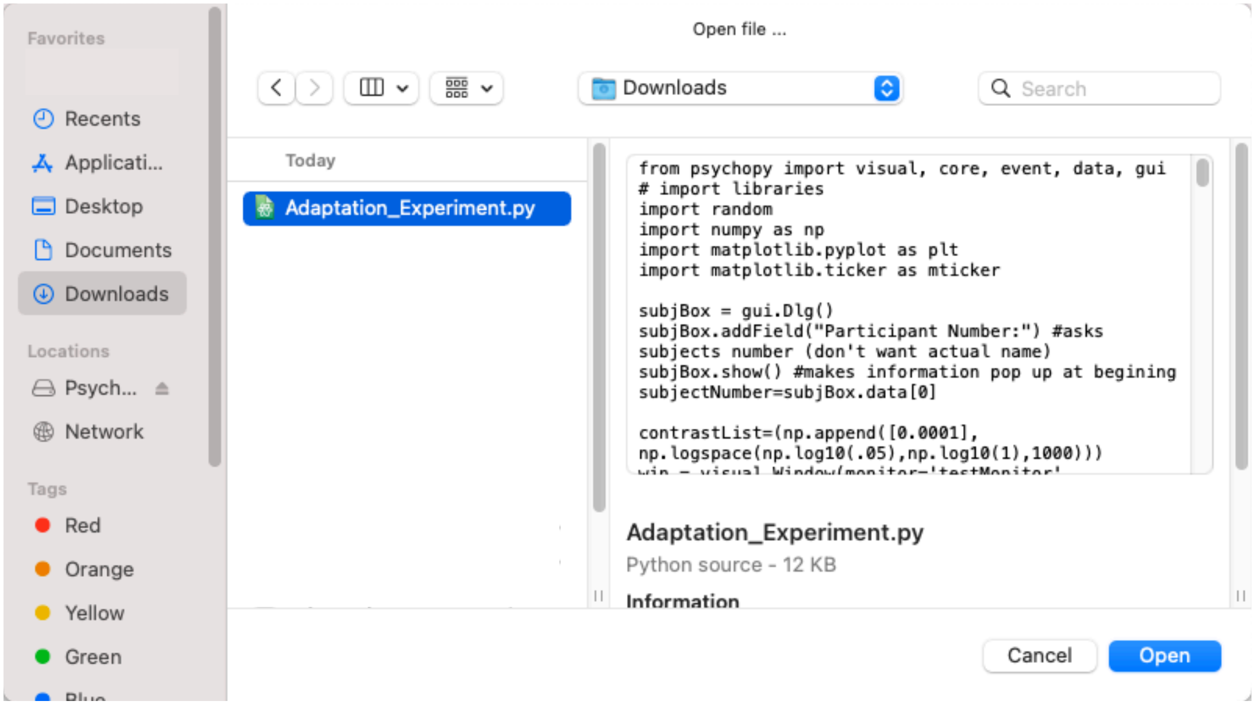

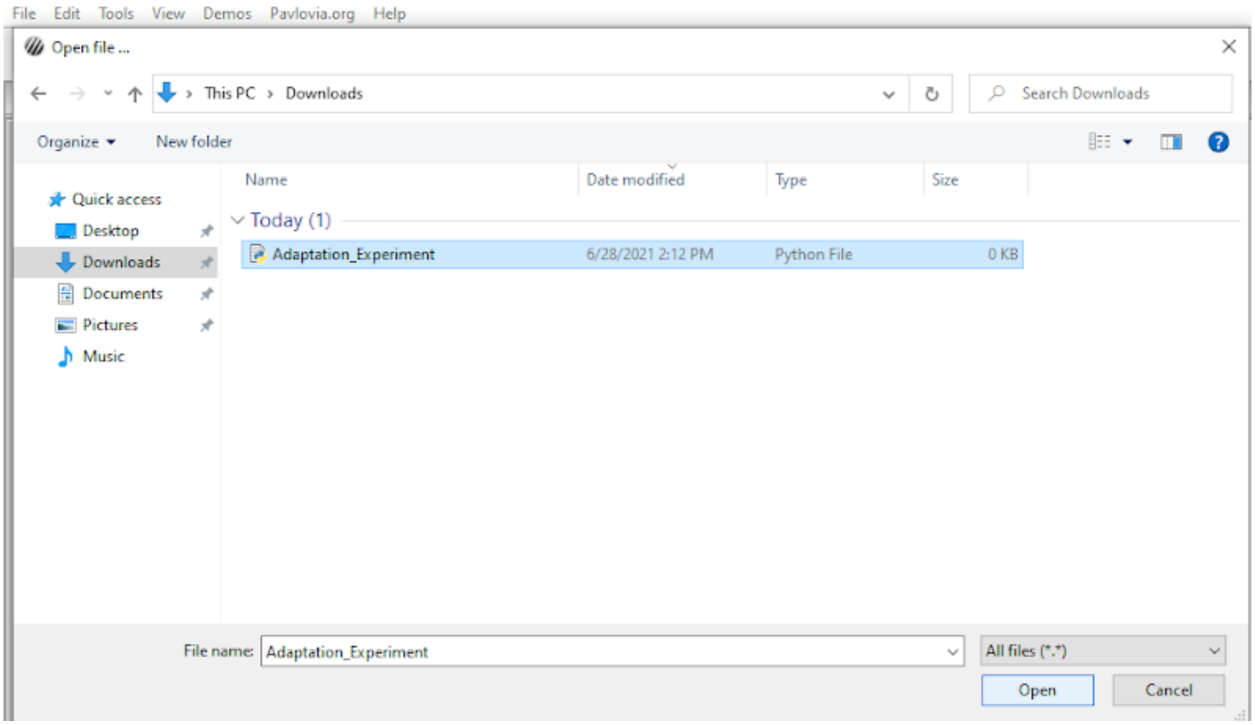

Mac and Windows Users

10. **Once the code is in PsychoPy, click the green "play" button to run the experiment**
11. **Follow the instructions provided in the experiment**
12. **Once the experiment ends and the window closes, reopen this PDF and continue reading on the next page**

Note: This PDF will not be available to you throughout the experiment.

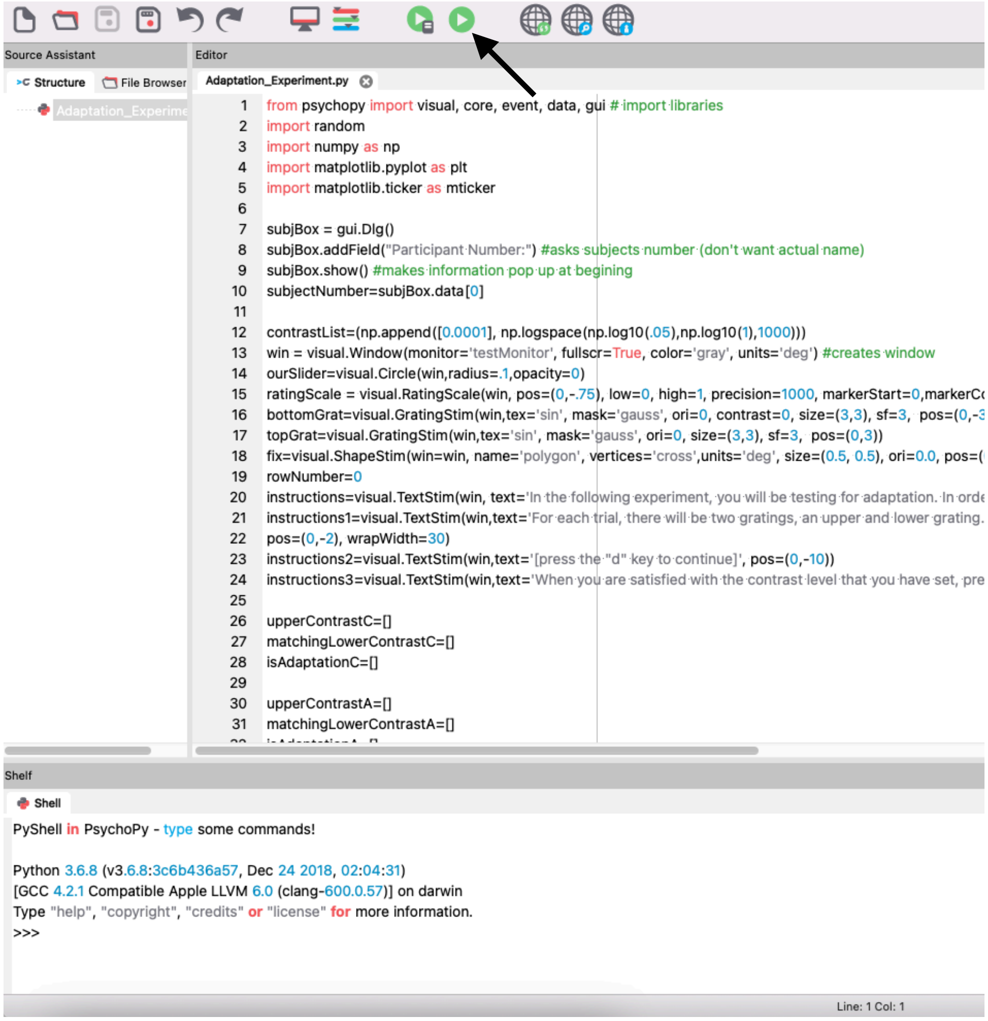

Mac Users

13. Now that you have completed the experiment, you can view your results
14. To access the results figure and data table, go to your downloads folder in Finder (or to whichever folder the code was saved to), and search the file names:

“Adaptation_Experiment_Participant(#)_DataTable”
“Adaptation_Experiment_Participant(#)_Figure”

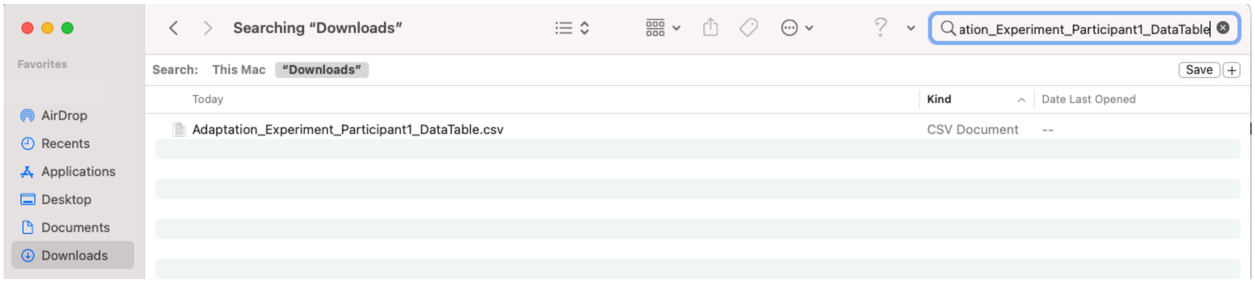

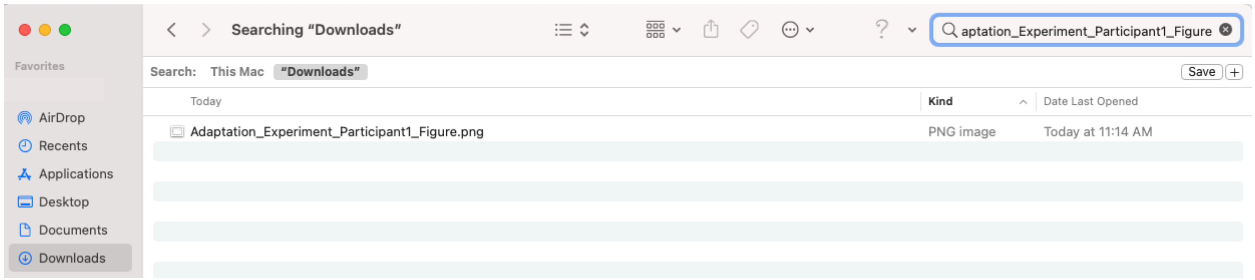

Windows Users

13. Now that you have completed the experiment, you can view your results
14. To access the results figure and data table, go to your downloads folder (or to whichever folder the code was saved to), and search the file names:

"Adaptation_Experiment_Participant(#)_DataTable"
"Adaptation_Experiment_Participant(#)_Figure"

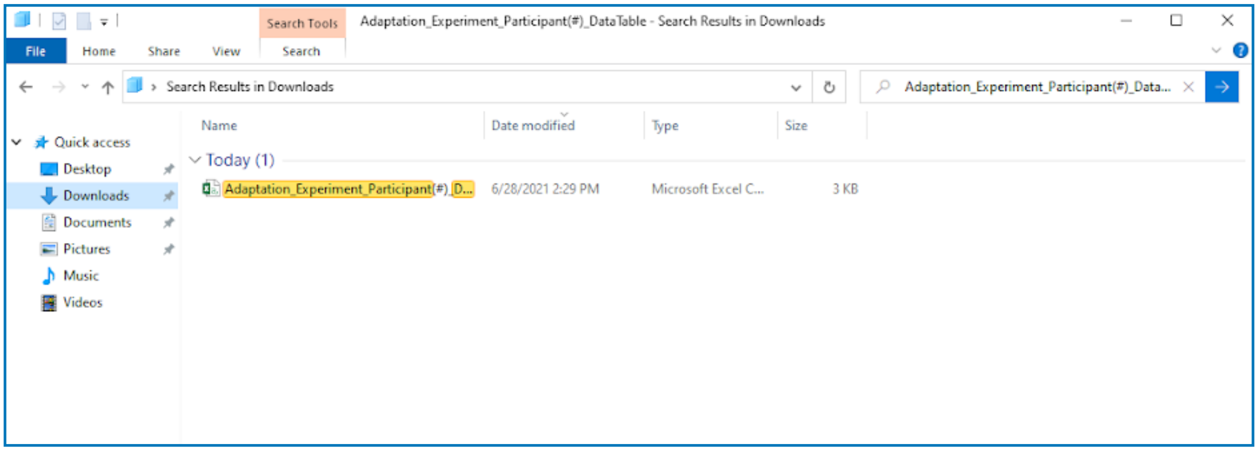

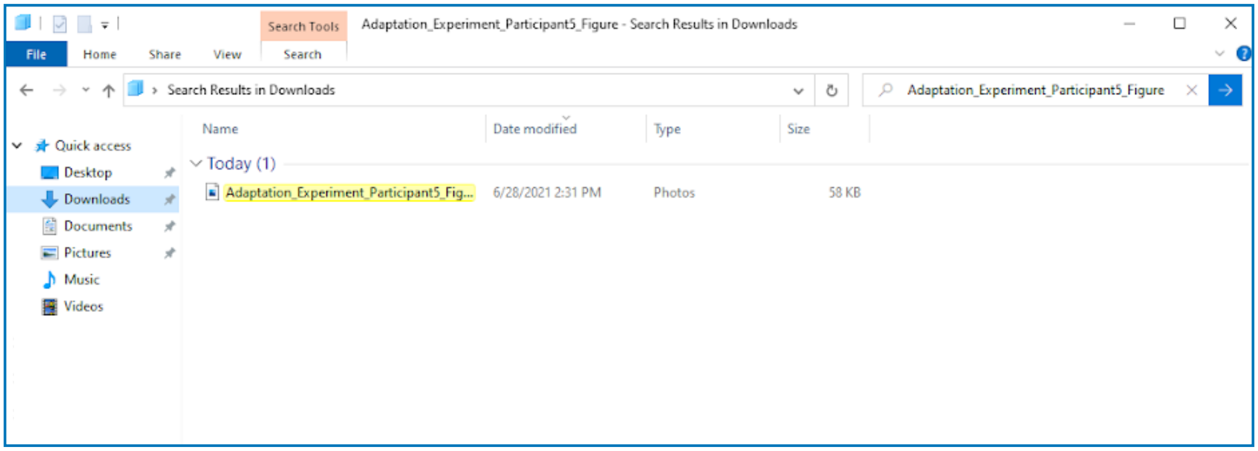

Supplement: Extended Data 2 — Active learning exercise code and instructions. Download Extended Data 2, ZIP file. [file enu-eN-CFN-0323-21-s02.zip › Instruction_Manual_Final.pdf]
